# Radiographic Angle-Based Machine Learning Models for the Diagnosis of Pes Planus and Pes Cavus: A Large-Scale Study Using Weight-Bearing Lateral Foot Radiographs

**DOI:** 10.3390/diagnostics16121929

**Published:** 2026-06-22

**Authors:** Rabia Taşdemir, Mustafa Işık, Ahmet Hakan İnce, Ebru Sena Poyraz, Şule Baysal, Ramazan Parıldar, Nevzat Gönder

**Affiliations:** 1 Department of Anatomy, Faculty of Medicine, Gaziantep Islam Science and Technology University, 27000 Gaziantep, Türkiye; 2 Department of Orthopedics and Traumatology, Faculty of Medicine, Gaziantep University, 27000 Gaziantep, Türkiye; 3 Electrical and Electronics Engineering, Scientific Research and Projects Coordination Unit, Gaziantep Islam Science and Technology University, 27000 Gaziantep, Türkiye; 4 Graduate School of Health Sciences, Gaziantep University, 27000 Gaziantep, Türkiye; 5 School of Medicine, Gaziantep Islam Science and Technology University, 27000 Gaziantep, Türkiye; 6 Department of Orthopedics and Traumatology, T.C. Ministry of Health 25 Aralık State Hospital, 27000 Gaziantep, Türkiye

**Keywords:** pes planus, pes cavus, calcaneal pitch angle, Meary angle, talar declination angle, machine learning

## Abstract

**Background/Objectives**: Pes planus and pes cavus are common foot deformities, which may lead to pain, functional limitations, and impairment of foot biomechanics. While calcaneal pitch, talar declination, and Meary angles, commonly used in diagnosis, provide objective information, their lack of a gold standard and the observer’s dependence on manual measurements limit their reliability. Therefore, in this study, these angles obtained from weight-bearing lateral foot radiographs were evaluated according to literature references, and the aim was to determine the model that provides the most accurate prediction in the diagnosis of pes planus using machine learning algorithms. It should be emphasized that, because the diagnostic labels were derived from literature-based thresholds of these same angles, the machine-learning task addressed here is the automated reproduction and standardization of expert, angle-threshold-based classification, rather than an independent clinical diagnosis from raw images. **Methods**: This retrospective study was conducted using weight-bearing lateral foot radiographs of 697 male patients obtained from the archives of public hospitals in Gaziantep. Calcaneal pitch, Meary angle, and talar declination angles were evaluated in both feet, and the data were labeled as normal, pes planus, and pes cavus. The dataset, consisting of a total of 1394 feet, was divided into training and test groups and analyzed using Random Forest, XGBoost, Logistic Regression, Support Vector Machine (SVM), and K-Nearest Neighbors (KNN) algorithms; the diagnostic performance of the models was compared using measures such as accuracy, F1 score, sensitivity, and specificity. **Results**: A total of 1394 feet from 697 male patients (mean age 24.8 ± 5.57 years) were analyzed using five machine learning algorithms with calcaneal pitch angle (CPA), Meary angle (MA), and talar declination angle (TDA) as reference labels. Ensemble-based methods showed superior performance, with XGBoost achieving perfect classification (Accuracy = 1.000) under all three labels for the left foot and 0.996–1.000 for the right foot, while Random Forest reached 0.986–1.000 across all experiments. Logistic Regression and SVM yielded moderate accuracies (0.905–0.973), whereas KNN consistently performed the weakest (0.905–0.964), particularly in the pes cavus subgroup. The near-perfect accuracy obtained when the labeling angle was itself included among the predictors reflects, at least in part, the algebraic reconstruction of the threshold rule from a same-source variable rather than genuine diagnostic generalization; results should therefore be interpreted with this in mind. **Conclusions**: This study demonstrates that machine learning, particularly ensemble methods such as XGBoost and Random Forest, provides high accuracy and consistency in diagnosing foot arch deformities based on radiographic angle measurements. Traditional models, such as Logistic Regression, still hold value in terms of clinical interpretability despite their lower performance. The findings suggest that machine learning-based approaches can offer objective, rapid, and reliable decision support tools for diagnosing pes planus and pes cavus, but external validation studies are necessary for clinical generalizability.

## 1. Introduction

Pes planus (flatfoot) is a common foot deformity characterized by a reduction or loss of the medial longitudinal arch [1,2]. Structural alterations such as excessive hindfoot valgus, forefoot abduction, calcaneal eversion, and medial rotation of the talus may also be observed in this deformity [1,2]. This condition may be congenital or acquired throughout life [1,2]. Clinical manifestations vary depending on the stage of the disorder, ranging from asymptomatic cases to severe deformities [1]. It has been reported that pes planus may disrupt lower extremity biomechanics, predispose individuals to secondary pathologies, cause pain and functional limitations, and adversely affect quality of life and foot function [1,3]. Therefore, accurate and reliable assessment of pes planus is clinically important. Pes cavus is characterized by varus in the hindfoot and an increased calcaneal slope. This reduces shock absorption and leads to eccentric loading, increasing the risk of fracture [4].

Various methods are used in the evaluation of pes planus and pes cavus, including radiological imaging [4,5], clinical examination, somatometric measurements [6], and digital footprint or plantar pressure analyses [5,6,7]. Among these, examination-based methods rely heavily on the examiner’s experience and may yield subjective results [6]. For this reason, angular measurements performed on weight-bearing lateral foot radiographs are among the preferred methods, particularly in the initial assessment of pes planus and pes cavus, as they provide an objective evaluation of hindfoot alignment and medial longitudinal arch height [3].

Commonly used angles in angular measurements on weight-bearing lateral foot radiographs include the calcaneal pitch, talar declination, and Meary’s angles [4,8]. The calcaneal pitch angle (calcaneal inclination angle) (CPA) is defined as the angle between the plantar surface of the calcaneus and a line drawn parallel to the ground, and provides information regarding the height of the medial longitudinal arch [9,10,11]. It is one of the most frequently used radiographic parameters in the evaluation of foot type, with reported normal values ranging from 18–30°. Below 18° is considered pes planus, and above 30° is considered pes cavus [9,10,12]. The talar declination angle (TDA) is the angle between the long axis of the talus and a line drawn parallel to the ground, reflecting plantar flexion and medial displacement of the talus. Normal values are generally considered to be approximately 18–24°, while values exceeding 30° are typically reported in individuals with pes planus. Below 18° is considered pes cavus [8,11]. Meary’s angle (the talo–first metatarsal angle) (MA) is formed between the long axis of the talus and the long axis of the first metatarsal, providing information regarding the alignment of the medial longitudinal arch. Angles between −4° and 4° are considered within the normal range; angles less than −4° are classified as pes planus, whereas angles greater than 4° are classified as pes cavus [4,8,11].

Although these angles are clinically valuable, the literature does not identify a single “gold standard angle” for foot type [2,5]. Moreover, there is no full consensus regarding the normal value ranges for arch height and radiographic angles [5]. Therefore, these angular parameters have been assessed either individually or collectively in many studies [13]. However, manual measurements may show interobserver variability, potentially leading to diagnostic discrepancies [14,15]. Additionally, manual assessment is time-consuming and may reduce diagnostic reliability in large sample sizes or routine clinical practice [16,17]. These limitations highlight the need for more objective, rapid, and reliable methods for the diagnosis of foot types.

In recent years, artificial intelligence and machine learning techniques have been increasingly applied to the automated analysis of radiographic images, enabling faster, more reliable, and more reproducible radiographic measurements [13]. Comparing the performance of different algorithms has become a major focus of current research to identify models that can be integrated into clinical practice [13]. Such approaches may minimize interobserver differences in manual measurements and enhance diagnostic accuracy.

Although numerous studies have evaluated pes planus using radiographic angle measurements, large-sample research systematically comparing these angles using machine learning algorithms is limited. In particular, investigating the three key angles (calcaneal pitch, talar declination, and Meary’s angles) separately to determine which algorithms yield the most accurate classification could contribute significantly to the diagnostic process.

Therefore, in our study, calcaneal pitch, talar declination, and Meary’s angles were measured on weight-bearing lateral foot radiographs, and foot types were identified based on reference values reported in the literature. These data were then applied to various machine learning algorithms to determine the model providing the highest predictive accuracy for pes planus and pes cavus diagnosis. It is important to state explicitly that the objective of the present work is not to perform an independent clinical diagnosis from raw radiographic images, but rather to automate and standardize the angle-threshold-based classification that experts already apply, and to identify which algorithm most faithfully and reproducibly replicates this expert labeling. This framing is essential for the correct interpretation of the performance metrics reported below.

## 2. Materials and Methods

### 2.1. Sample Selection

The study was conducted between 16 March 2025 and 26 September 2025, in accordance with the principles of the Declaration of Helsinki.

For the study, radiographs stored in the archives of various public hospitals in Gaziantep, Turkey, the majority of which were taken during medical examinations for military conscription, were retrospectively scanned and images were chosen based on inclusion and exclusion criteria. Radiographs of both feet were obtained from 697 patients who met the criteria. All images were of males, with an average age of 24.80 ± 5.57 years.

### 2.2. Inclusion Criteria

This study included images of individuals aged 10–60 years old who did not have any condition that prevented walking (such as hemiplegia, paraplegia, or hip and knee prostheses) and who had not undergone foot surgery.

### 2.3. Exclusion Criteria

Images with motion artifacts in the examination area, images belonging to individuals with systemic inflammatory disease, and images of individuals with congenital structural defects that could affect lower extremity alignment were not included in the study.

### 2.4. Measurement of Angles

**Figure 1:**
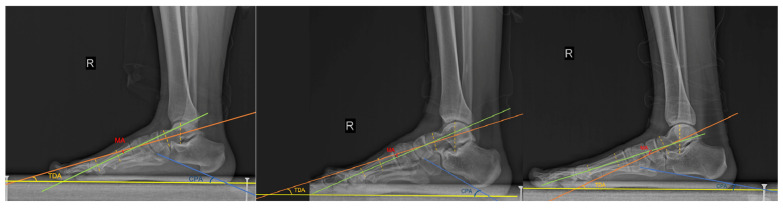
Illustration of angular measurements on weight-bearing lateral radiographs showing (**left**) a normal foot, (**middle**) a pes cavus foot, and (**right**) the pes planus foot. The yellow line represents the ground-parallel reference line. The orange line indicates the longitudinal axis of the talus. The green line denotes the longitudinal axis of the first metatarsal. The blue line represents the line tangential to the inferior surface of the calcaneus. Meary’s angle (MA) is defined as the angle between the talar axis and the first metatarsal axis. The talar declination angle (TDA) is defined as the angle between the talar axis and the ground-parallel reference line. The calcaneal pitch angle (CPA) is defined as the angle between the line tangential to the inferior surface of the calcaneus and the ground-parallel reference line.

In this study, a total of 697 participants were evaluated to investigate foot arch conditions through quantitative radiographic measurements. For each participant, both the left and right feet were assessed, and three clinically relevant angular parameters were recorded: the Calcaneal Pitch Angle (CPA), Meary’s Angle (MA), and the Talar Declination Angle (TDA) (Figure 1).

These measurements are widely recognized in the literature as indicators of foot arch morphology and are commonly used for identifying normal arches, pes planus (flatfoot), and pes cavus (high-arched foot) [8]. Prior to the primary measurements, a reliability study was conducted to assess inter-rater consistency. Two independent raters (Anatomist RT with 7 years of experience and orthopedist NG with 7 years of experience) each measured CPA, MA, and TDA of 50 randomly selected right and 50 left feet twice in a test–retest format. Inter-observer repeatability of the angles was evaluated using intraclass correlation coefficients (ICCs). For both feet, an ICC value was calculated. The results demonstrated excellent reliability: right foot CPA ICC = 0.91, MA ICC = 0.88, TDA ICC = 0.89, left foot CPA ICC = 0.93, MA ICC = 0.90, TDA ICC = 0.85. The technical error of measurement (TEM) for all raters was <0.001 for feet angle. These results indicate that the angle measurements were reliably obtained. The reported coefficients are inter-rater (inter-observer) ICCs, estimated with a two-way random-effects model for absolute agreement, single measures, corresponding to the ICC(2,1) form in the Shrout and Fleiss notation; intra-rater (test–retest) reliability was additionally examined and was of comparable magnitude. The technical error of measurement was calculated in the unit of the measurements themselves, i.e., in degrees (°); the reported value of <0.001 refers to the relative TEM (coefficient of variation). All measurements were performed by an experienced orthopedist and were repeated twice for both feet. The average of the two measurements was recorded.

### 2.5. Statistical Analysis

Statistical analyses were performed using SPSS version 27.0 (IBM Corp., Armonk, NY, USA). Descriptive statistics for continuous variables are presented as mean ± standard deviation, while categorical variables are expressed as frequencies and percentages. Statistical significance was set at *p* < 0.05.

### 2.6. Data Preparation and Model Development

The collected data provided a comprehensive set of observations across 1394 feet (697 left and 697 right), enabling a balanced investigation of both sides. Expert annotations were included in the dataset to serve as ground-truth labels, classifying each foot as normal (0), pes planus (1), or pes cavus (2). These labels formed the basis for supervised machine learning approaches. Although a total of 1394 feet (697 left and 697 right) were available, the machine learning analyses were conducted separately for the left and right feet. Left-foot models were trained and evaluated exclusively using left-foot measurements (CPA, MA, and TDA; n = 697), whereas right-foot models were developed using only right-foot measurements (n = 697). Therefore, left and right feet were not merged into a single machine learning dataset, and each experiment was performed independently for the corresponding side. The present study employed a feature-based machine learning approach rather than an image-based classification framework. The input variables consisted of three radiographic angle measurements obtained from the corresponding foot: CPA), (MA), and (TDA). These quantitative measurements were used as predictor variables, while the expert-defined foot classifications (normal, pes planus, and pes cavus) served as the target labels. No raw radiographic images or image-derived features were used during model training. Each foot was labeled independently according to literature-based threshold values of the selected reference angle. Therefore, the unit of analysis in this study was the foot rather than the participant.

To analyze the predictive capacity of the measured angles, the dataset was initially partitioned into training (70%) and testing (30%) subsets using a stratified sampling strategy to preserve the proportional distribution of the classes. Model development and internal validation were performed exclusively on the training subset using stratified 5-fold cross-validation. This procedure was employed to assess model stability, reduce the risk of overfitting, and enhance generalizability. Following cross-validation, the final models were evaluated on the independent test set. Several machine learning algorithms namely Random Forest, XGBoost, Logistic Regression, Support Vector Machine (SVM), and K-Nearest Neighbors (KNN) were implemented and systematically compared. Prior to model training, standard preprocessing steps, including handling of missing values and feature scaling, were applied to ensure consistency and robustness of the results.

Model performance was then evaluated using accuracy, balanced accuracy, averaged F1 score, precision, and recall, along with confusion matrices to provide class-specific insights. This methodological framework establishes a rigorous basis for assessing the diagnostic utility of machine learning techniques in detecting pes planus and pes cavus from radiographic angular measurements.

## 3. Results

Descriptive statistics for age and measured parameters for the study are given in Table 1.

### 3.1. Left Foot Experiments

Table 2 shows the distribution of the frequency of normal foot, pes planus, and pes cavus in left foot radiographs (according to the reference values of each angle).

#### 3.1.1. CPA-Based Pes Planus Label (Left Foot)

In the first experiment, the classification of pes planus for the left foot was performed by taking the CPA-based annotation as the reference label (*PesPlanusNo0,Yes1 LEFT*). The CPA is a widely utilized angular measurement in clinical radiology, providing a direct indication of the alignment and inclination of the calcaneus relative to the foot arch. Accordingly, it was selected as the ground-truth label in this stage of the study. The predictive features used to train the machine learning models included the measured values of left CPA, MA, and TDA, allowing for the evaluation of how well these combined angular parameters can replicate the expert-defined CPA-based classification. This setup provided a baseline framework for comparing the diagnostic efficiency of different algorithms in identifying normal arches, pes planus, and pes cavus based on CPA reference labeling.

The Table 3 summarizes the comparative performance of five machine learning algorithms applied to the left foot dataset using CPA-based pes planus labels as the reference standard. Among the models, XGBoost achieved perfect performance across all metrics (Accuracy, Balanced Accuracy, Precision, Recall, and F1), indicating its strong capability to capture the underlying patterns of the dataset. However, such flawless results may also suggest a potential risk of overfitting, which should be considered when interpreting the findings. The Random Forest model demonstrated similarly high accuracy (98.6%) and F1 (92.0%), correctly identifying most cases of normal and pes planus conditions, though minor misclassifications were observed in the pes cavus class. Logistic Regression and SVM (RBF) achieved slightly lower overall accuracy (~90%), yet their balanced accuracy values (96.0% and 91.6%, respectively) highlight their ability to more fairly represent all classes, including the minority pes cavus category. Finally, KNN performed moderately well (Accuracy 93.8%), but its lower balanced accuracy (84.9%) suggests some difficulty in handling class imbalance. Overall, the results emphasize that while tree-based ensemble methods (Random Forest and XGBoost) provided the highest predictive power, linear and distance-based models contributed valuable complementary insights by maintaining stronger class balance, particularly for underrepresented categories.

**Figure 2:**
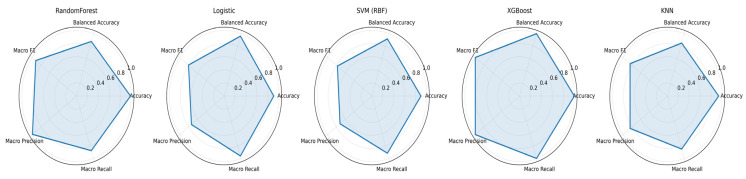
Radar plots of model performance on left foot CPA-labeled dataset.

Figure 2 Radar plots comparing the classification performance of five machine learning algorithms (Random Forest, Logistic Regression, Support Vector Machine, XGBoost, and K-Nearest Neighbors) for the left foot CPA-labeled dataset across multiple evaluation metrics (Accuracy, Balanced Accuracy, F1, Precision, and Recall). The visual representation highlights that XGBoost and Random Forest achieved the most consistent and superior performance across all metrics, while Logistic Regression and SVM showed moderate yet competitive results. In contrast, KNN exhibited relatively lower precision and recall, indicating a tendency toward misclassification in certain cases. Overall, the radar charts emphasize the robustness of ensemble-based models (RF and XGBoost) in handling CPA-based pes planus classification for the left foot.

#### 3.1.2. MA-Based Pes Planus Label (Left Foot)

In this section, Meary’s angle of the left foot was employed as the primary radiographic reference to classify pes planus (PesPlanusML). Three angular measurements, CPA (left), MA (left), and TDA (left), were used as predictive features, and classification was conducted using five machine learning algorithms: Random Forest, XGBoost, Logistic Regression, Support Vector Machine (SVM), and K-Nearest Neighbors (KNN). The dataset, comprising 697 participants, was divided into 70% training and 30% testing subsets to ensure robust evaluation. Model performance was assessed using multiple complementary metrics, including Accuracy, Balanced Accuracy, F1, Precision, and Recall, along with confusion matrices to provide a detailed understanding of misclassification patterns. This comprehensive analysis enables direct comparison of traditional classifiers with ensemble-based methods, offering insights into the relative strengths and weaknesses of each approach when Meary’s angle serves as the diagnostic reference standard.

Table 4 shows the classification performance of five machine learning algorithms using Meary’s angle (left foot) as the pes planus reference label (PesPlanusML). The dataset was divided into 70% training and 30% testing subsets. The results show that XGBoost achieved perfect performance across all metrics (1.000), which demonstrates strong predictive ability but also suggests a potential risk of overfitting. Random Forest exhibited similarly high performance (Accuracy ≈ 0.999), indicating robust generalization. Logistic Regression and SVM reached moderate but consistent scores, while KNN yielded the lowest performance, particularly in recall (0.884), reflecting limited sensitivity in identifying pes planus cases. Overall, ensemble-based methods, particularly tree-based models, demonstrated the most reliable classification outcomes.

**Figure 3:**
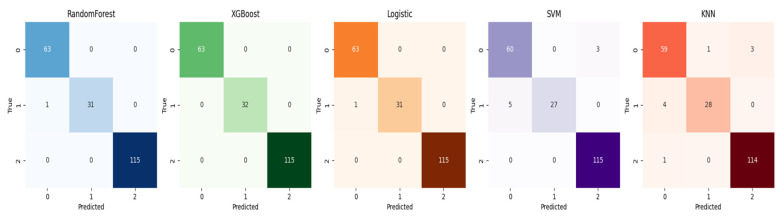
Confusion matrices for pes planus classification based on Meary’s angle (left foot).

Figure 3 shows the confusion matrices of five machine learning algorithms (Random Forest, XGBoost, Logistic Regression, SVM, and KNN) for pes planus classification using Meary’s angle (left foot) as the reference label (PesPlanusML). The results demonstrate that XGBoost achieved a perfect classification, correctly identifying all cases without misclassification, whereas Random Forest produced nearly identical results with only a single misclassification. Logistic Regression also performed strongly, showing minimal errors. In contrast, SVM and KNN displayed a higher number of misclassifications, particularly in borderline cases, leading to reduced sensitivity in detecting pes planus. These findings reinforce the superior capability of tree-based ensemble methods, while also highlighting potential weaknesses of distance- and margin-based classifiers in this clinical classification task.

#### 3.1.3. TDA-Based Pes Planus Label (Left Foot)

In this stage of the study, the talar declination angle (TDA) of the left foot was employed as the diagnostic reference (PesPlanusTDAL). Unlike the CPA and MA, which primarily reflect longitudinal arch alignment, the TDA provides a direct assessment of hindfoot orientation and is particularly sensitive to variations in flatfoot severity. This characteristic makes it a valuable criterion for evaluating pes planus in clinical practice. The classification task was carried out using left CPA, left MA, and left TDA as predictors. The focus here was to examine how well machine learning algorithms can replicate the expert-defined labeling based on TDA, and whether ensemble methods or conventional classifiers perform more reliably under this alternative reference framework.

Table 5 shows that when the TDA of the left foot was used as the reference label, the distribution of results across the five classifiers revealed a clear performance gradient. XGBoost once again reached ceiling values across all metrics, perfectly replicating the expert-based labeling. While this can be interpreted as evidence of strong model data alignment, it also calls for caution, as such flawless fitting may not generalize equally well in unseen populations. Random Forest, with accuracy above 99%, proved nearly indistinguishable from XGBoost, yet its slightly lower recall suggests it may miss occasional positive cases. By contrast, Logistic Regression and SVM achieved results in the mid-90% range, representing stable but less dominant alternatives that may offer more transparency and lower risk of overfitting. KNN, although exceeding 91% accuracy, consistently lagged behind, highlighting the limitations of distance-based classifiers in capturing the structural complexity of radiographic features. Taken together, the TDA-based analysis reinforces the strength of ensemble learners, while also showing that linear models can provide reasonably balanced outputs, especially when interpretability is prioritized over raw predictive power.

**Figure 4:**
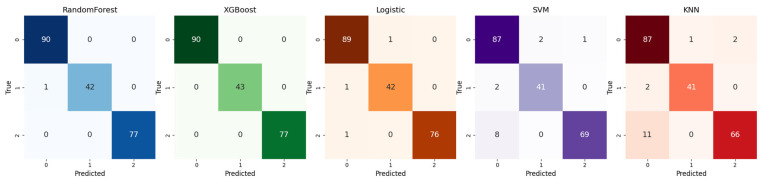
Confusion matrices for pes planus classification based on TDA.

Figure 4 shows the confusion matrices of five machine learning algorithms for pes planus classification using the TDA of the left foot as the reference label (PesPlanusTDAL). The visual comparison illustrates distinct error patterns across models. XGBoost produced flawless separation, with all cases correctly assigned to their true categories, while Random Forest showed nearly identical results, with misclassifications limited to a negligible fraction. Logistic Regression and SVM, although not achieving perfect predictions, displayed consistent accuracy with only a small number of errors, particularly in borderline instances. In contrast, KNN generated a noticeably higher number of incorrect classifications, underscoring its difficulty in handling the complex feature interactions underlying radiographic measurements. These confusion matrices highlight not only the dominance of ensemble-based methods but also the trade-offs between transparency, computational complexity, and sensitivity across different algorithmic families.

As shown in Table 6, the 5-fold cross-validation results were highly consistent across folds, indicating stable model performance. XGBoost and Random Forest achieved the best overall results, with both models maintaining near-perfect accuracy and balanced accuracy values. Logistic Regression and SVM yielded slightly lower classification performance but remained effective across all classes. The relatively small standard deviations observed for all metrics demonstrate that the proposed models were not substantially affected by variations in data partitioning, further supporting the robustness of the findings.

### 3.2. Right Foot Experiments

Table 7 shows the frequency of normal foot, pes planus, and pes cavus according to angle measurements made on right foot radiographs.

#### 3.2.1. CPA-Based Pes Planus Label (Right Foot)

In this experiment, the CPA of the right foot was adopted as the reference label for pes planus classification. The CPA is a widely accepted radiographic indicator of calcaneal alignment and foot arch structure. While the left foot analysis provided a baseline framework, incorporating the right foot allows for a more comprehensive evaluation of bilateral variations and potential asymmetries in pes planus diagnosis. The predictive features used in this setup were Right CPA, Right MA, and Right TDA, and classification was carried out with five supervised learning algorithms: Random Forest, XGBoost, Logistic Regression, Support Vector Machine (SVM), and K-Nearest Neighbors (KNN). The dataset was stratified into 70% training and 30% testing subsets to maintain class proportions across both normal and pes planus cases. Model performance was assessed through Accuracy, Balanced Accuracy, F1, Precision, and Recall, supplemented by confusion matrices to capture detailed misclassification patterns. This right-foot analysis provides an opportunity to directly compare outcomes with the left-foot results, thereby highlighting whether diagnostic consistency is preserved across both sides or if clinically relevant discrepancies emerge.

The results which is shown Table 8 obtained using the right foot CPA-based labeling demonstrate that both XGBoost and Random Forest achieve performance close to perfection, with accuracy levels above 99% and F1 scores above 0.98. These findings confirm the strong suitability of ensemble learners for handling radiographic features in pes planus detection. In contrast, while Logistic Regression and SVM reached accuracy levels around 97%, their relatively lower balanced accuracy values (0.89–0.90) suggest that they are less effective in handling class distribution differences, particularly in minority categories. KNN, although yielding competitive overall accuracy (96.4%), lagged slightly behind in recall, highlighting its limitations in sensitivity. Overall, the CPA right-foot analysis illustrates a clear hierarchy of models, where ensemble-based methods lead, linear and margin-based models provide stable yet less balanced performance, and distance-based approaches show constraints in generalization.

**Figure 5:**
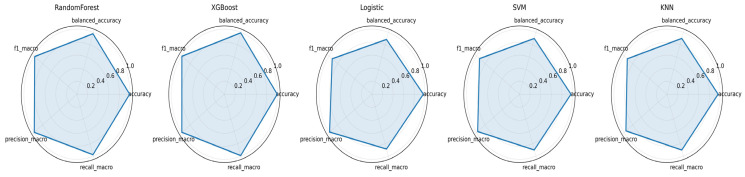
Radar plots of model performance on right foot CPA-labeled dataset.

Figure 5 shows the radar plots illustrating the performance of five machine learning algorithms for CPA-based pes planus classification of the right foot. The visualization reveals that XGBoost and Random Forest occupy almost the entire performance area, confirming their dominance across all evaluation criteria. Logistic Regression and SVM, while slightly behind, maintain relatively balanced profiles, with strong precision yet reduced balance in recall. KNN shows a more contracted radar shape, particularly along the recall and balanced accuracy axes, indicating that its predictions are less stable across classes. The overall shape comparison emphasizes how ensemble models consistently preserve both sensitivity and precision, whereas linear and distance-based methods tend to trade off balance for accuracy in this diagnostic setting.

**Figure 6:**
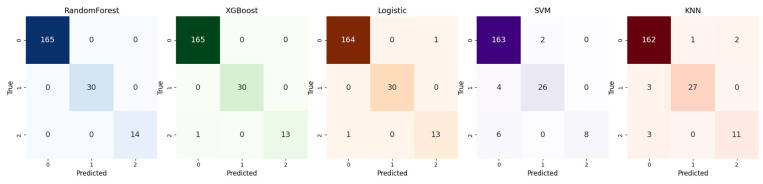
Confusion matrices for right foot CPA-based pes planus.

The results shown in Figure 6 demonstrate that XGBoost and Random Forest produced nearly flawless separations, with almost all cases correctly assigned, including the minority pes cavus group. Logistic Regression also delivered highly accurate predictions, with only isolated errors in the cavus class. SVM showed slightly weaker stability, misclassifying a few more cases from the pes cavus and pes planus categories, though overall accuracy remained high. By contrast, KNN exhibited a more dispersed error distribution, particularly misclassifying cavus cases as normal or planus, which points to its sensitivity to class imbalance and boundary overlap. These comparative patterns highlight how ensemble approaches maintain dominance in predictive reliability, while distance-based classifiers like KNN struggle to preserve consistency in multi-class medical datasets.

**Figure 7:**
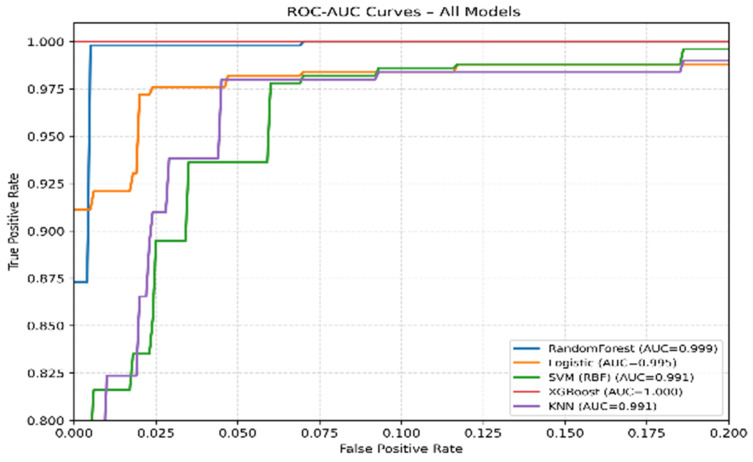
ROC–AUC curves of the evaluated ML models.

**Figure 8:**
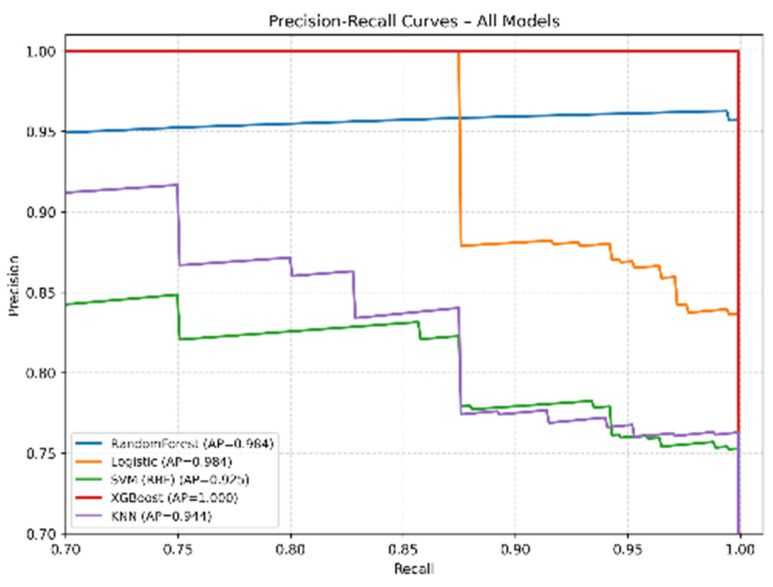
Precision–Recall curves of the evaluated ML models.

Figure 7 presents the ROC–AUC curves of the evaluated machine learning models. All classifiers demonstrated excellent discriminative performance, with AUC values greater than 0.99. XGBoost achieved the highest performance (AUC = 1.000), followed by Random Forest (AUC = 0.999), while Logistic Regression, SVM, and KNN also showed strong classification capability. The concentration of the ROC curves near the upper-left corner indicates high true positive rates and low false positive rates across different classification thresholds. Figure 8 presents the Precision–Recall (PR) curves of the same models. XGBoost achieved the highest Average Precision (AP = 1.000), followed by Random Forest and Logistic Regression (AP = 0.984). Although SVM (AP = 0.925) and KNN (AP = 0.944) exhibited comparatively lower performance, they still maintained high precision across a wide range of recall values. Overall, both ROC–AUC and PR analyses confirmed the strong predictive performance of the evaluated machine learning models for foot arch classification.

#### 3.2.2. MA-Based Pes Planus Label (Right Foot)

For the right foot analysis, Meary’s angle was adopted as the principal reference criterion for determining flatfoot status. Unlike the left-side evaluation, this stage was intended to explore whether the diagnostic patterns observed previously also extend to the contralateral foot, and to examine potential asymmetry in clinical presentation. The analysis incorporated three angular descriptors from the right foot, CPA, MA, and TDA, as explanatory variables. A panel of five supervised learning methods (Random Forest, XGBoost, Logistic Regression, Support Vector Machine, and K-Nearest Neighbors) was employed to capture the predictive relationship between these radiographic measures and the expert-defined categories in Table 9. To maintain proportional representation of the classes, the dataset was randomly divided into training and test partitions in a 70–30 ratio. Evaluation of the models was based not only on global metrics such as accuracy and F1, but also on fairness-sensitive indicators like balanced accuracy and recall, complemented with confusion matrices to visualize the distribution of errors. This configuration allowed for a nuanced appraisal of each algorithm’s reliability when Meary’s angle serves as the guiding standard for right-foot pes planus classification.

The comparative outcomes reveal clear differentiation among the classifiers. Random Forest and XGBoost achieved flawless scores across all metrics, indicating that both algorithms captured the dataset’s structure with exceptional precision. However, such perfect alignment also signals the need for cautious interpretation, as it may reflect potential overfitting when applied beyond the current sample. Logistic Regression performed consistently well, with an accuracy close to 97% and the highest balanced accuracy among the non-ensemble models, showing its strength in handling class distribution. SVM produced slightly lower but still robust results (≈96% accuracy), reflecting a stable margin-based approach with a relatively even error distribution. KNN, while delivering acceptable results (≈95% accuracy), trailed behind the others, highlighting the challenges of distance-based methods in modeling subtle radiographic distinctions. Taken together, the right-foot MA-based analysis underscores the supremacy of ensemble methods, while also confirming that linear and margin-based classifiers can provide reliable yet less dominant alternatives.

**Figure 9:**
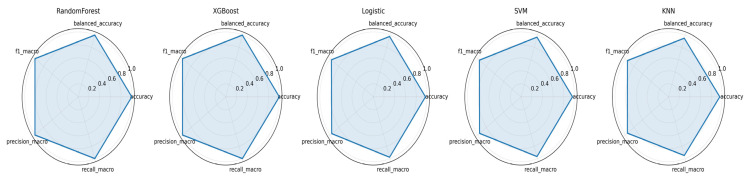
Radar plots of model performance on right foot MA-labeled dataset.

Figure 9 presents radar visualizations of the five classifiers when Meary’s angle of the right foot served as the reference label. The star-shaped profiles highlight that Random Forest and XGBoost almost completely fill the metric space, reflecting exceptionally stable predictions across all evaluation dimensions. Logistic Regression displays a slightly more compact but still well-balanced shape, suggesting that it achieves a strong trade-off between sensitivity and precision. SVM, although achieving a relatively uniform profile, shows subtle contractions along the recall axis, indicating that some borderline cases were harder to detect. By contrast, KNN exhibits the most irregular radar shape, with noticeable dips in balanced accuracy and recall, illustrating that its predictions are more vulnerable to class distribution effects. These visual patterns collectively illustrate not only the dominance of tree-based ensemble methods but also the distinct error tendencies of linear, margin-based, and distance-based approaches when evaluated against Meary’s angle.

**Figure 10:**
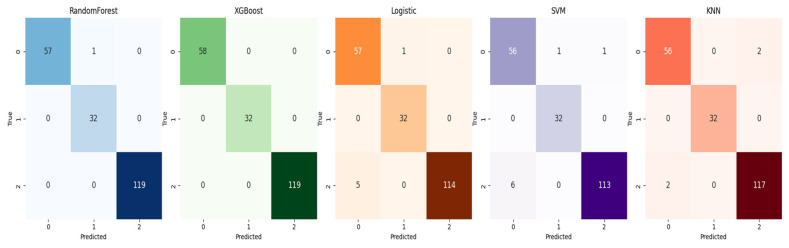
Confusion matrices of five classifiers for right foot MA-labeled dataset.

Figure 10 illustrates the confusion matrices for the five algorithms when Meary’s angle (right foot) was adopted as the labeling criterion. Both Random Forest and XGBoost show almost perfect separations, with only a single misclassification across all categories, underscoring their consistency in handling class boundaries. Logistic Regression achieved high accuracy overall, but a small number of cavus cases were incorrectly identified, indicating limited sensitivity for this minority class. SVM produced a more noticeable spread of errors, particularly by misassigning several cavus cases as normal arches, reflecting challenges in margin-based decision boundaries. KNN also displayed scattered misclassifications, with errors affecting both normal and cavus groups, highlighting that its distance-driven predictions are more vulnerable to overlap between classes. Taken together, these error patterns reveal how ensemble methods provide not only superior accuracy but also resilience in detecting less frequent categories, whereas linear, margin-based, and neighborhood-based models exhibit specific weaknesses in classifying atypical foot arch configurations.

#### 3.2.3. TDA-Based Pes Planus Label (Right Foot)

In the final stage of the right-foot analysis, the TDA was used as the primary diagnostic label. This angle, reflecting the alignment between the talus and calcaneus in the sagittal plane, provides an alternative radiographic perspective compared to CPA and MA. By incorporating TDA as the ground-truth label, the experiment was designed to evaluate how effectively computational models can replicate expert-based classifications using this measurement. For this purpose, the predictive set again comprised the three angular features of the right foot (Right CPA, Right MA, Right TDA). Five machine learning algorithms, Random Forest, XGBoost, Logistic Regression, SVM, and KNN, were applied under a stratified 70–30 train–test partitioning in Table 10. Model performance was assessed using multiple evaluation metrics (accuracy, balanced accuracy, F1, precision, and recall), complemented with radar visualizations and confusion matrices to capture not only the overall predictive strength but also the class-specific error tendencies. This setup allows for direct comparison with earlier right-foot experiments and highlights the diagnostic value of TDA relative to other angular measures.

The evaluation based on the TDA right-foot reference demonstrates striking contrasts among the algorithms. Random Forest and XGBoost yielded nearly indistinguishable results, both approaching perfect accuracy and stability across all metrics, underlining their exceptional robustness for this diagnostic marker. In comparison, Logistic Regression maintained a solid balance between precision and recall, achieving around 95% accuracy and indicating reliable generalization to different foot arch categories. SVM, while still competitive, displayed a more modest profile, with accuracy settling near 92%, suggesting that its margin-based structure was less adaptive to the distribution of TDA values. KNN ranked lowest in this setting, particularly in balanced accuracy (0.892), revealing its sensitivity to overlapping boundaries among classes. Collectively, these outcomes emphasize that tree-based ensembles remain dominant when TDA is employed as the labeling criterion, while linear and distance-driven models, though functional, face clear limitations in capturing the full complexity of right-foot angular measurements.

**Figure 11:**
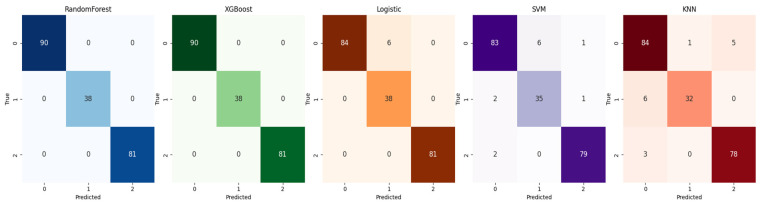
Confusion matrices of five classifiers for right foot TDA-labeled dataset.

Figure 11 displays the misclassification patterns of the five algorithms when the Talar Declination Angle (TDA) of the right foot was used as the ground-truth label. Both Random Forest and XGBoost produced almost identical and highly reliable outcomes, with all classes separated cleanly and virtually no overlap, demonstrating that ensemble learners can capture the structural distinctions embedded in the TDA measurement. Logistic Regression, although achieving respectable accuracy, introduced visible errors in the normal and cavus categories, suggesting that its linear decision boundaries may fail to accommodate the curved separations implied by this angle. SVM revealed further instability, particularly in the cavus group, where several cases were reassigned to the normal category, reflecting limitations in handling subtle angular variations. KNN, while operational, generated the broadest spread of errors, with misclassifications affecting all three categories, underscoring the vulnerability of distance-based models when class boundaries are not sharply defined. These matrices highlight that although all approaches can approximate TDA-based labeling, ensemble frameworks remain the most consistent in preserving class integrity.

**Figure 12:**
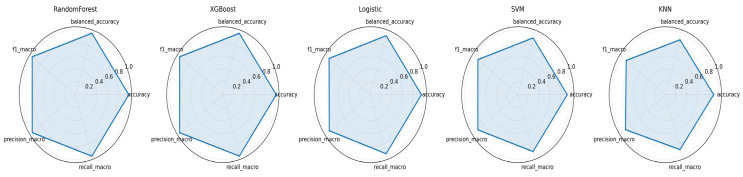
Radar plots of five classifiers for right foot TDA-Refereced dataset.

Figure 12 depicts the radar profiles of the five algorithms when the Talar Declination Angle (TDA) was applied as the labeling criterion for the right foot. The figures reveal that Random Forest and XGBoost create nearly full pentagonal shapes, pointing to highly uniform performance across all evaluation metrics and suggesting that these ensemble models adapt seamlessly to the complexity of the TDA-based categorization. Logistic Regression, while not reaching the same scale, maintains a relatively smooth contour, with strong precision but slightly contracted values for recall, indicating that its linear formulation handles the majority of cases well but may miss subtler class distinctions. SVM produces a balanced yet more compressed polygon, showing that its margin-based approach remains reliable but less expansive than ensembles. By contrast, KNN displays the most asymmetrical shape, with noticeable narrowing along the balanced accuracy and recall axes, highlighting its vulnerability to class overlap. Collectively, these visual patterns stress that while multiple algorithms achieve competitive results, the geometry of the radar plots clearly distinguishes the ensembles as the most consistent performers under TDA-based annotation.

As shown in Table 11, to further evaluate model robustness, stratified 5-fold cross-validation was performed. The cross-validation results were highly consistent with those obtained from the independent holdout test set. XGBoost achieved the best overall performance with an accuracy of 0.9957 ± 0.0058 and a balanced accuracy of 0.9914 ± 0.0144, followed closely by Random Forest (accuracy: 0.9942 ± 0.0054). Logistic Regression and SVM showed slightly lower overall accuracy but maintained high balanced accuracy values (>0.96), indicating stable performance across classes. The low standard deviations observed across folds suggest that the models produced consistent results and were not overly sensitive to data partitioning.

## 4. Discussion

A common diagnosis of pes planus cannot be made by measuring MA, CPA, and TDA together on lateral radiographs. The main reason for this is that these methods can produce inconsistent results with each other [18]. The reasons for the inconsistency are that pes planus deformities can occur in a single region or multiple regions of the foot, in different planes (sagittal, coronal, etc.). On the other hand, while MA and CPA focus on the collapse of the medial longitudinal arch, 3 parameters evaluate different anatomical alignments of the foot. This leads to different parameters giving different results in the same radiographic image [15,19].

This study comprehensively evaluated the diagnostic performance of machine learning algorithms in classifying pes planus and pes cavus using three different radiographic angular measurements (CPA, MA, TDA). The findings consistently demonstrated that tree-based ensemble methods, specifically XGBoost and Random Forest, achieved exceptional performance across all experiments, regardless of the reference angle or foot (left or right). Their near-perfect accuracy (often above 99%), F1 scores, and balanced accuracy underscore a superior capacity to capture the complex, non-linear relationships inherent in radiographic foot arch data. However, the flawless performance of XGBoost in multiple experiments raises an important question regarding potential overfitting. The models’ perfect alignment with the dataset may indicate they have learned the specific patterns of the current sample too well, which could compromise their generalizability to unseen populations or different clinical settings. Therefore, external validation on independent, multi-center datasets is a crucial next step before any clinical deployment can be considered.

The exceptionally high classification performance observed in several experiments, including accuracy values approaching 1.000, should be interpreted within the context of the study design. While these findings demonstrate the strong predictive capabilities of the evaluated models, they are also influenced by the characteristics of the dataset and the way the reference labels were defined. The feature set was limited to three radiographic measurements (CPA, MA, and TDA), all of which are closely associated with foot arch morphology and exhibit relatively low variability in measurement. In addition, the target labels were assigned according to established threshold values reported in the literature and derived from these same angular measurements.

As a result, some classification tasks inherently involved predicting labels that were directly linked to one of the input variables. For example, in the CPA-based experiments, CPA was used both as a predictor and as the criterion for assigning class labels. Under such conditions, tree-based ensemble algorithms, particularly XGBoost and Random Forest, are well suited to identify and reproduce these threshold-driven decision rules through successive data partitioning. Consequently, the near-perfect performance observed in these models is not unexpected, as the algorithms can effectively learn and replicate the underlying classification criteria. Therefore, these results should be viewed primarily as evidence of the models’ ability to automate and reproduce established radiographic classification frameworks rather than as proof of flawless clinical diagnostic capability.

Although stratified 5-fold cross-validation was employed during model development and final performance was assessed using an independent holdout test set, caution is still warranted when interpreting the findings. The study population was obtained from a single center and represented a relatively homogeneous cohort. Accordingly, further validation using larger and more diverse populations from multiple institutions is necessary before the proposed models can be considered for broader clinical application.

Conversely, traditional classifiers like Logistic Regression and SVM delivered moderate yet clinically acceptable performance (90–97% accuracy). Their relatively strong balanced accuracy scores suggest a more robust handling of class distribution compared to their overall accuracy might imply. Their key advantage lies in their greater interpretability; the decision boundaries of Logistic Regression, for instance, can offer clinicians transparent insights into which angular measurements most significantly drive the classification, fostering trust and understanding. As expected, the K-Nearest Neighbors (KNN) algorithm underperformed relative to its counterparts. Its lower balanced accuracy and recall, particularly for the pes cavus class, highlight its limitations in managing class imbalance and its susceptibility to ambiguous class boundaries in complex feature spaces, which are common in medical diagnostic tasks.

The lack of significant performance asymmetry between the left and right foot analyses reinforces the reliability of the proposed models for bilateral assessment. Furthermore, the fact that all three angular parameters (CPA, MA, TDA) could effectively serve as ground-truth labels confirms their utility as complementary and robust indicators of foot arch morphology, allowing for flexibility in clinical application depending on the preferred radiographic measurement.

In the literature review, pes planus diagnosis has mostly been performed using deep learning methods on radiographic images [20,21,22,23,24]. It has been reported that a stepwise evolutionary neural network (CNN and FlatNet) based deep learning model on radiographic images shows a higher accuracy and reliability rate than human observers [23,24]. We did not come across any studies in which pes planus was predicted using machine learning algorithms with angle methods.

Our findings should also be positioned against the most recent state-of-the-art. Noh et al. reported a deep-learning system that automatically measures flatfoot angles on weight-bearing lateral radiographs, while Ryu et al. introduced a cascaded convolutional neural network (FlatNet) for automated landmark identification and angle measurement. More recently, Devnath et al. proposed an explainable, cost-efficient pipeline that extracts deep features with a modified VGG16 backbone, reduces them through statistical feature selection, and classifies pes planus with conventional machine-learning models, reporting an AUC of 0.99 (95% CI 0.96–1.00) for Random Forest together with decision-curve analysis and LIME-based explainability [15,24,25]. Two aspects distinguish those studies from ours and are important for fair interpretation. First, the deep-learning approaches learn directly from image pixels and validate against an external or held-out patient cohort, so their reported accuracy reflects genuine image-based discrimination; in contrast, our models operate on pre-computed angles whose thresholds also define the labels, which inflates the apparent accuracy as discussed above. Second, those studies report threshold-independent metrics (AUC) and, in the case of Devnath et al., explainability and decision-curve analysis; we have therefore added ROC/AUC and precision–recall analyses (Figure 7 and Figure 8) so that our results can be benchmarked on more than accuracy alone.

The strengths of our study include the ability to more clearly identify which parameter influences the outcome or diagnosis using machine learning algorithms based on angles. The interpretability of these results is more advantageous in clinical trials. Furthermore, while larger datasets provide more reliable results for deep learning models, machine learning algorithms can achieve more reliable results with less data in geographically challenging areas where image collection is difficult. Another point is that angle measurement protocols are defined and standardized in the literature. Therefore, similar results can be obtained in different locations. However, image quality, image contrast, and device differences in the images used for deep learning can significantly affect the results. Finally, machine learning algorithms are easier, faster, and more practical to implement. They can be easily applied in locations without a graphics processing unit (GPU) or complex software infrastructure.

The limitations of our study include the fact that our sample consisted of individuals of the same gender and within a narrow age range, which restricted our ability to demonstrate gender differences and track changes in foot anatomy with age. The retrospective nature of the study also prevented the identification of confounding factors. The radiographs used in the study were obtained only from hospitals in Gaziantep city center, specifically images taken within the framework of military recruitment protocols. Since the sample may not fully represent the Turkish population, this could limit the generalizability of the model. Beyond these points, the most important limitation is methodological: because the labels were defined by thresholding the same angles that were used as predictors, the very high accuracies primarily reflect the reconstruction of these threshold rules rather than independent diagnostic performance, and they should not be extrapolated to image-based or clinical diagnosis. The labels themselves are surrogate, single-angle definitions made in the absence of an accepted gold standard, so any model can only be as valid as the reference threshold it reproduces. The wide age span (10–60 years) is a further limitation, since arch morphology and the radiographic angles may differ between, for example, a 10-year-old and a 60-year-old foot; although a formal age-stratified subgroup analysis was limited by the available sample, this remains an important direction, and age-specific reference values should ideally be used. The exclusively male, military-conscription-based, single-center sample further restricts generalisability across sex and age, and no external validation cohort was available. In terms of clinical significance, the practical value of this work lies less in the headline accuracy figures and more in providing a transparent, rapid, and reproducible means of standardizing angle-based foot-type labeling, reducing inter-observer variability in routine reporting, and serving as a low-resource decision-support adjunct in settings without the infrastructure required for deep-learning image analysis; confirmation of genuine diagnostic utility, however, will require image-based models and prospective, multi-center external validation against a clinical reference standard. Finally, because the present analysis relied solely on the three numerical angular features and did not incorporate the radiographic images themselves, future studies should pursue a multimodal approach that combines image-derived features with these angular measurements; such integration of all feature types is expected to provide genuine, non-circular diagnostic information beyond the angle-threshold rules and to move the task from automated labeling towards true image-based diagnosis.

## 5. Conclusions

This research successfully establishes the potent utility of machine learning, and specifically ensemble methods, for the automated and accurate diagnosis of foot arch conditions from standard radiographic angles. The results lead to several definitive conclusions. Firstly, ensemble learners, particularly XGBoost and Random Forest, are the most powerful classifiers for this task, delivering unrivaled predictive accuracy and consistency. Secondly, while less dominant, traditional models like Logistic Regression remain valuable, offering a compelling trade-off between acceptable performance and model interpretability, which is vital in a clinical context. Finally, the study underscores the necessity of rigorous external validation to confirm the generalizability of these promising results and mitigate overfitting concerns. In summary, this work provides a robust methodological framework and compelling evidence that machine learning-driven tools, especially those based on ensemble techniques, can significantly augment clinical decision-making by providing objective, rapid, and highly accurate assessments of pes planus and pes cavus.

## Figures and Tables

**Table 1:** Descriptive statistics of age and angles.

Parameters	Minimum	Maximum	Mean ± SD
Age	13.00	53.00	24.8 ± 5.57
LCPA	3.80	39.10	20.1 ± 5.39
RCPA	4.50	40.70	21.22 ± 5.79
LMA	−26.10	22.00	−4.52 ± 8.48
RMA	−26.90	25.10	−5.24 ± 8.66
LTDA	−17.00	40.20	19.79 ± 5.65
RTDA	−17.00	16.40	19.54 ± 7.90

LCPA-RCPA: Left and Right Calcaneal Pitch Angle, LMA-RMA: Left and Right Meary’s Angle, LTDA-RTDA: Left and Right Talar Declination Angle, SD: standard deviation.

**Table 2:** Frequency of normal foot, pes planus, and pes cavus according to angle measurements in radiographs of the left foot.

Parameters	Normal Foot N (%)	Pes Planus N (%)	Pes Cavus N (%)
CPA	555 (79.6%)	117 (16.8%)	25 (3.6%)
MA	209 (30.0%)	105 (15.1%)	383 (54.9%)
TDA	299 (42.9%)	143 (20.5%)	255 (36.6%)

CPA: Calcaneal Pitch Angle, MA: Meary’s Angle, TDA: Talar Declination Angle, N: count, %: percentage.

**Table 3:** Model Performance Comparison for CPA-Based Pes Planus (Left Foot).

Model	Accuracy	Balanced Accuracy	F1	Precision	Recall
Random Forest	0.9857	0.8750	0.9201	0.9941	0.8750
Logistic Regression	0.9048	0.9601	0.8050	0.7388	0.9601
SVM (RBF)	0.8952	0.9164	0.7782	0.7190	0.9164
XGBoost	1.0000	1.0000	1.0000	1.0000	1.0000
KNN	0.9381	0.8495	0.8401	0.8399	0.8495

**Table 4:** Classification performance (Left MA—PesPlanusML).

Model	Accuracy	Balanced Accuracy	F1	Precision	Recall
Random Forest	0.998571	0.996825	0.997590	0.998450	0.996825
XGBoost	1.000000	1.000000	1.000000	1.000000	1.000000
Logistic Regression	0.952610	0.931241	0.941850	0.954774	0.931241
SVM	0.935416	0.914230	0.921259	0.930914	0.914230
KNN	0.919620	0.883810	0.896651	0.916133	0.883810

**Table 5:** Classification performance (TDA Left—PesPlanusTDAL).

Model	Accuracy	Balanced Accuracy	F1	Precision	Recall
Random Forest	0.9928	0.9892	0.9917	0.9945	0.9892
XGBoost	1.0000	1.0000	1.0000	1.0000	1.0000
Logistic	0.9627	0.9560	0.9604	0.9664	0.9560
SVM	0.9469	0.9415	0.9465	0.9531	0.9415
KNN	0.9182	0.9171	0.9205	0.9255	0.9171

**Table 6:** 5-Fold Cross-Validation Results Classification (Left Foot).

Model	Accuracy	Balanced Accuracy	F1-Score	Precision	Recall
Random Forest	0.9957 ± 0.0035	0.9600 ± 0.0327	0.9769 ± 0.0189	0.9982 ± 0.0015	0.9600 ± 0.0327
Logistic Regression	0.9240 ± 0.0321	0.9682 ± 0.0135	0.8432 ± 0.0393	0.7792 ± 0.0463	0.9682 ± 0.0135
SVM (RBF)	0.9154 ± 0.0260	0.9497 ± 0.0291	0.8348 ± 0.0279	0.7731 ± 0.0369	0.9497 ± 0.0291
XGBoost	0.9986 ± 0.0029	0.9867 ± 0.0267	0.9923 ± 0.0154	0.9994 ± 0.0012	0.9867 ± 0.0267
KNN	0.9684 ± 0.0086	0.8857 ± 0.0624	0.9138 ± 0.0387	0.9597 ± 0.0402	0.8857 ± 0.0624

**Table 7:** Distribution of the frequency of normal foot, pes planus, and pes cavus according to angle measurements in right foot radiographs.

Parameters	Normal Foot N (%)	Pes Planus N (%)	Pes Cavus N (%)
CPA	551 (79.1%)	101 (14.5%)	45 (6.5%)
MA	192 (27.5%)	396 (56.8%)	109 (15.6%)
TDA	300 (43%)	269 (38.6%)	128 (18.4%)

CPA: Calcaneal Pitch Angle, MA: Meary’s Angle, TDA: Talar Declination Angle, N: count, %: percentage.

**Table 8:** Classification performance (Right CPA).

Model	Accuracy	Balanced Accuracy	F1	Precision	Recall
Random Forest	0.9942	0.9772	0.9849	0.9950	0.9772
XGBoost	0.9957	0.9914	0.9920	0.9933	0.9914
Logistic Regression	0.9698	0.8855	0.9270	0.9878	0.8855
SVM	0.9712	0.9009	0.9312	0.9747	0.9009
KNN	0.9640	0.9018	0.9258	0.9578	0.9018

**Table 9:** Classification performance (Meary Right).

Model	Accuracy	Balanced Accuracy	F1	Precision	Recall
Random Forest	1.0000	1.0000	1.0000	1.0000	1.0000
XGBoost	1.0000	1.0000	1.0000	1.0000	1.0000
Logistic Regression	0.9727	0.9783	0.9697	0.9628	0.9783
SVM	0.9626	0.9670	0.9606	0.9562	0.9670
KNN	0.9569	0.9499	0.9496	0.9508	0.9499

**Table 10:** Classification performance (Reference Right TDA).

Model	Accuracy	Balanced Accuracy	F1	Precision	Recall
Random Forest	0.9986	0.9989	0.9981	0.9974	0.9989
XGBoost	0.9986	0.9974	0.9981	0.9989	0.9974
Logistic Regression	0.9554	0.9597	0.9532	0.9484	0.9597
SVM	0.9253	0.9238	0.9230	0.9241	0.9238
KNN	0.9052	0.8922	0.9024	0.9205	0.8922

**Table 11:** 5-Fold Cross-Validation Results for the Right Foot.

Model	Accuracy	Balanced Accuracy	F1	Precision	Recall
Random Forest	0.9942 ± 0.0054	0.9704 ± 0.0277	0.9826 ± 0.0166	0.9976 ± 0.0022	0.9704 ± 0.0277
Logistic Regression	0.9311 ± 0.0228	0.9683 ± 0.0137	0.8822 ± 0.0373	0.8294 ± 0.0473	0.9683 ± 0.0137
SVM (RBF)	0.9239 ± 0.0154	0.9679 ± 0.0065	0.8715 ± 0.0213	0.8123 ± 0.0261	0.9679 ± 0.0065
XGBoost	0.9957 ± 0.0058	0.9914 ± 0.0144	0.9920 ± 0.0098	0.9933 ± 0.0119	0.9914 ± 0.0144
KNN	0.9626 ± 0.0116	0.9013 ± 0.0460	0.9225 ± 0.0294	0.9492 ± 0.0208	0.9013 ± 0.0460

## Data Availability

The data that support the findings of this study are available on request from the corresponding author. The data are not publicly available due to privacy or ethical restrictions.

## Abbreviations

The following abbreviations are used in this manuscript:

AI	Artificial Intelligence
CNN	Convolutional Neural Network
CPA	Calcaneal Pitch Angle
CT	Computed Tomography
GPU	Graphics Processing Unit
ICC	Intraclass Correlation Coefficient
KNN	K-Nearest Neighbors
LCPA	Left Calcaneal Pitch Angle
LMA	Left Meary’s Angle
LTDA	Left Talar Declination Angle
MA	Meary’s Angle
ML	Machine Learning
MRI	Magnetic Resonance Imaging
RCPA	Right Calcaneal Pitch Angle
RF	Random Forest
RMA	Right Meary’s Angle
RTDA	Right Talar Declination Angle
SD	Standard Deviation
SPSS	Statistical Package for the Social Sciences
SVM	Support Vector Machine
TDA	Talar Declination Angle
TEM	Technical Error of Measurement
XGBoost	Extreme Gradient Boosting
